# Supplementary material for: Isolation of Salvia miltiorrhiza Kaurene Synthase-like (KSL) Gene Promoter and Its Regulation by Ethephon and Yeast Extract
Source: Genes (Basel). 2022 Dec 24;14(1):54. doi: 10.3390/genes14010054 (PMC9859234; doi:10.3390/genes14010054)
Supplement: Supplementary file 1 [file genes-14-00054-s001.zip › Description to Supplement material.pdf]

File S1. Complete list of putative *cis*-active elements found within obtained *SmKSL* promoter by PlantPAN3.0.

Table S1. Transcription factors and other proteins co-expressed with *A. thaliana KSL* gene (At1g79460; *AtKSL*) identified by Expression Angler software.

Table S2. Seventeen *trans*-factor genes co-expressed with *AT1G79460* within the r range 0.7-1.0.

Table S3. Quantitative effect of YE and ET on the *SmKSL* gene expression. Mean values of gene expression changes found by RT-PCR analysis were applied.

Table S4. Interaction between YE and ET during the Tt biosynthesis.
