# Supplementary material for: Isolation of Salvia miltiorrhiza Kaurene Synthase-like (KSL) Gene Promoter and Its Regulation by Ethephon and Yeast Extract
Source: Genes (Basel). 2022 Dec 24;14(1):54. doi: 10.3390/genes14010054 (PMC9859234; doi:10.3390/genes14010054)
Supplement: Supplementary file 1 [file genes-14-00054-s001.zip › Table S1.pdf]

Tab. S1

Transcription factors and other proteins co-expressed with *A. thaliana KSL* gene (At1g79460; *AtKSL*) identified by Expression Angler software.

GeneID	WGCNA Correlation*	Symbol	Alias	Description
AT1G79460	1.0000	GA2	ATKS1	ARABIDOPSIS THALIANA ENT-KAURENE SYNTHASE 1
AT5G43990	0.0612	SUVR2	--	SET-domain containing protein lysine methyltransferase family protein
AT1G59540	0.0607	ZCF125	--	P-loop containing nucleoside triphosphate hydrolases superfamily protein
AT5G37010	0.0595	--	--	rhoGTPase-activating protein
AT5G05620	0.0577	GCP2	ATGCP2	ARABIDOPSIS THALIANA GAMMA-TUBULIN COMPLEX
AT3G18524	0.0557	MSH2	ATMSH2	MUTS homolog 2
AT1G09450	0.0556	Haspin	AtHaspin	Protein kinase superfamily protein
AT2G07170	0.0552	--	--	ARM repeat superfamily protein
AT4G14330	0.0552	--	--	P-loop containing nucleoside triphosphate hydrolases superfamily protein
AT3G61650	0.0551	TUBG1	--	gamma-tubulin
AT2G32590	0.0541	EMB2795	--	Condensin complex subunit
AT2G42120	0.0540	POLD2	--	DNA polymerase delta small subunit
AT3G26050	0.0535	--	--	TPX2 (targeting protein for Xklp2) protein family
AT5G10080	0.0532	--	--	Eukaryotic aspartylprotease family protein
AT4G21270	0.0530	ATK1	KATAP	KINESIN-LIKE PROTEIN IN ARABIDOPSIS THALIAN
AT2G47230	0.0530	DUF6	ATDUF6	DOMAIN OF UNKNOWN FUNCTION 724 6
AT4G29360	0.0528	--	--	O-Glycosylhydrolases family 17 protein

AT1G03830	0.0527	--	--	guanylate-binding family protein
AT3G20260	0.0527	--	--	DUF1666 family protein (DUF1666)
AT4G26760	0.0523	MAP65-2	--	microtubule-associated protein 65-2

The "WGCNA Correlation*" kolumn refer to the connection strength between two genes. This value is between 0 and 1, and the higher value refers to a stronger connection or co-expression of genes.
