# Supplementary material for: Isolation of Salvia miltiorrhiza Kaurene Synthase-like (KSL) Gene Promoter and Its Regulation by Ethephon and Yeast Extract
Source: Genes (Basel). 2022 Dec 24;14(1):54. doi: 10.3390/genes14010054 (PMC9859234; doi:10.3390/genes14010054)
Supplement: Supplementary file 1 [file genes-14-00054-s001.zip › Table S2.pdf]

Tab. S2. Seventeen *trans*-factor genes co-expressed with AT1G79460 within the r range 0.7-1.0.

Nr	Locus	Encoded transcription factor	Co-expression coefficient (r)	Putative function
**AtGenExpress Pathogen Compendium**				
1	**At5g38140**	NF-YC12; nu clear factor Y, subunit C12	0.706	Subunit of the NF-YC12, CCAAT-binding factor (CBF)
2	At2g24645	Transcription factor, B3 family protein	0.712	-
3	At1g73870	BBX16; COL7; B-box type zinc finger protein with CCT domain	0.713	Light-induced anthocyanin accumulation
4	At1g49560	HHO6; Homeodomain-like superfamily protein	0.725	-
5	At3g13040	gammaMYB2; myb-like HTH transcriptional regulator family protein	0.727	-
6	At2g39900	WLIM2a;_GATA type zinc finger transcription factor family protein	0.740	Predominantly expressed in pollen. Regulates actin cytoskeleton organization.
7	At5g07690	ATMYB29; MYB29; PMG2; RAO7; myb domain protein 29	0.751	Encodes a putative transcription factor (MYB29) that acts as a negative regulator of mitochondria stress responses.
8	At3g46780	PTAC16; plastid transcriptionally active 16	0.769	Circadian clock regulation.
9	At5g61420	AtMYB28; HAG1; MYB28; PMG1; myb domain protein 28	0.771	Positive regulation of aliphatic glucosinolate biosynthesis. Expression is induced by touch, wounding and glucose.
10	At1g65260	PTAC4;VIPP1; plastid transcriptionally active 4	0.774	Encodes a protein required for thylakoid membrane formation.
11	At1g06040	BBX24; B-boxzincfinger family protein	0.775	Photomorphogenesis.
12	At5g18410	ATSRA1, PIR121	0.780	Actin nucleation, trichome morphogenesis.
13	**At5g65310**	ATHB5; HB5 homeobox protein 5	0.825	Positive regulator of ABA-responsiveness, mediating the inhibitory effect of ABA on growth during seedling establishment.
14	At4g12980	Auxin-responsive family protein	0.840	Activated by OXS2 under the treatment of salt.
15	At4g18390	TCP2; TEOSINTE BRANCHED 1, cycloidea and PCF transcription factor 2	0.877	Transcription factor regulating photomorphogenic growth by activating HY5 and HYH.
**AtGenExpress Hormone and Chemical Compendium**				
1	AT5G50915	CKG, cytokinin-responsive growth regulator	0.711	Member of the bHLH family of transcription factors. Acts within a cytokinin signaling pathway to promote cell proliferation and

				expansion.
2	**At2g27070**	ARR13; RR13 response regulator 13	0.734	Response to auxin.
**AtGenExpress Abiotic Stress Compendium**				
none				
