# Supplementary material for: Isolation of Salvia miltiorrhiza Kaurene Synthase-like (KSL) Gene Promoter and Its Regulation by Ethephon and Yeast Extract
Source: Genes (Basel). 2022 Dec 24;14(1):54. doi: 10.3390/genes14010054 (PMC9859234; doi:10.3390/genes14010054)
Supplement: Supplementary file 1 [file genes-14-00054-s001.zip › Table S3.pdf]

Table S3
Quantitative effect of YE and ET on the *SmKSL* gene expression. Mean values of gene expression changes found by RT-PCR analysis were applied. Dominant mode of YE and ET interaction is antagonism, only three samples written in bold indicated synergistic relation.

Time [hrs]	24	48	72
ET 0.05 mM + YE 0.5 %	0.27	1.45	1.71
ET 0.05 mM	2.34	2.37	1.02
YE 0.5 %	0.24	1.93	3.00
Sum: ET 0.05 mM + YE 0.5 %	2.58	4.34	4.02
Effect:	antagonistic	antagonistic	antagonistic
ET 0.10 mM + YE 0.5 %	0.52	25.85	0.26
ET 0.10 mM	0.12	12.73	1.29
YE 0.5 %	0.24	1.93	3.00
Sum: ET 0.10 mM + YE 0.5 %	0.36	14.70	3.29
Effect:	**synergy**	**synergy**	antagonistic
ET 0.25 mM + YE 0.5 %	0.15	9.12	2.85
ET 0.25 mM	0.17	0.77	1.79
YE 0.5 %	0.24	1.93	3.00
Sum: ET 0.25 mM + YE 0.5 %	0.51	2.70	3.79
Effect:	antagonistic	**synergy**	antagonistic
ET 0.50 mM + YE 0.5 %	0.28	0.87	1.66
ET 0.50 mM	0.30	0.29	3.63
YE 0.5 %	0.24	1.93	3.00
Sum: ET 0.50 mM + YE 0.5 %	0.54	2.22	6.63
Effect:	antagonistic	antagonistic	antagonistic
