# Supplementary material for: Isolation of Salvia miltiorrhiza Kaurene Synthase-like (KSL) Gene Promoter and Its Regulation by Ethephon and Yeast Extract
Source: Genes (Basel). 2022 Dec 24;14(1):54. doi: 10.3390/genes14010054 (PMC9859234; doi:10.3390/genes14010054)
Supplement: Supplementary file 1 [file genes-14-00054-s001.zip › Table S4.pdf]

Table S4. Interaction between YE 0.5 % and ET (0.05, 0.10, 0.25, 0.50 mM) occurring during Tt biosynthesis. The interaction was defined as described in Material and Methods. Mean values of Tt concentration calculated after the separate application of YE 0.5 % and ET (0.05, 0.10, 0.25, 0.50 mM) were compared with results obtained for YE 0.5 % and ET (0.05, 0.10, 0.25, 0.50 mM) used together. Presented results suggest the strong antagonistic interaction between YE 0.5% and ET (0.05, 0.10, 0.25, 0.50 mM) in the course of Tt biosynthesis. Only two samples marked in bold showed a weak synergy, while the antagonistic effects are common and much stronger, suggesting that they represent the true nature of YE 0.5% and ET (0.05, 0.10, 0.25, 0.50 mM) interaction during the Tt biosynthesis. Antag. indicates antagonistic, while Syn. is the synergistic interaction.

Applied elicitors and their interaction	Mean concentration of Tt [mg g^{-1} DW] in the function of time [days]					
	10	20	30	40	50	60
YE 0.5 % + ET 0.05 mM	0.12	1.61	2.21	7.14	8.20	4.61
YE 0.5 %	0.26	1.48	4.20	10.56	13.30	11.82
ET 0.05 mM	0.04	0.01	0.01	0.01	0.01	0.01
Sum: YE 0.5 % and ET 0.05 mM	0.30	1.49	4.21	10.57	13.31	11.83
Interaction type	0.12<0.30<br>Antag.	**1.61>1.49<br>Syn.**	2.21<4.21<br>Antag.	7.14<10.57<br>Antag.	8.20<13.31<br>Antag.	4.61<11.83<br>Antag.
YE 0.5 % + ET 0.10mM	0.38	0.96	0.59	3.51	4.33	1.00
YE 0.5 %	0.26	1.48	4.20	10.56	13.30	11.82
ET 0.10mM	0.01	0.01	0.02	0.07	0.06	0.42
Sum: YE 0.5 % and ET 0.10mM	0.27	1.49	4.22	10.63	13.36	12.24
Interaction type	**0.38>0.27<br>Syn.**	0.96<1.49<br>Antag.	0.59<4.22<br>Antag.	3.51<10.63<br>Antag.	4.33<13.36<br>Antag.	1.00<12.24<br>Antag.
YE 0.5 % + ET 0.25 mM	0.00	1.32	0.00	1.16	0.05	0.05
YE 0.5 %	0.26	1.48	4.20	10.56	13.30	11.82
ET 0.25 mM	0.15	0.02	0.00	0.00	0.20	0.22
Sum: YE 0.5 % and ET 0.25 mM	0.41	1.50	4.20	10.56	13.50	12.04
Interaction type	0.00<0.41<br>Antag.	1.32<1.50<br>Antag.	0.00<4.20<br>Antag.	1.16<10.56<br>Antag.	0.05<13.50<br>Antag.	0.05<12.04<br>Antag.
YE 0.5 % + ET 0.50mM	0.18	0.36	3.06	3.49	0.93	0.76
YE 0.5 %	0.26	1.48	4.20	10.56	13.30	11.82
ET 0.50mM	0.11	0.67	0.19	0.26	0.03	0.02
Sum: YE 0.5 % and ET 0.50mM	0.37	2.15	4.39	10.82	13.33	11.84

Interaction type	0.18<0.37 Antag.	0.36<2.15 Antag.	3.06<4.39 Antag.	3.49<10.82 Antag.	0.93<13.33 Antag.	0.76<11.84 Antag.
